# HPV-16 Infection Is Associated with a High Content of CD39 and CD73 Ectonucleotidases in Cervical Samples from Patients with CIN-1

**DOI:** 10.1155/2019/4651627

**Published:** 2019-05-07

**Authors:** María de Lourdes Mora-García, Sofía López-Cisneros, Vianey Gutiérrez-Serrano, Rosario García-Rocha, Benny Weiss-Steider, Jorge Hernández-Montes, Héctor I. Sánchez-Peña, Luis Roberto Ávila-Ibarra, Christian Azucena Don-López, Ricardo Muñóz-Godínez, Daniela Berenice Torres Pineda, Rommel Chacón-Salinas, Luis Vallejo-Castillo, Sonia Mayra Pérez-Tapia, Alberto Monroy-García

**Affiliations:** ^1^Laboratorio de Inmunobiología, UIDCC-UMIEZ, FES-Zaragoza, UNAM, Ciudad de México, Mexico; ^2^Laboratorio de Inmunología y Cáncer, Unidad de Investigación Médica en Enfermedades Oncológicas, CMN SXXI, Instituto Mexicano del Seguro Social, Ciudad de México, Mexico; ^3^Departamento de Ginecología, HGZ No. 2-A Troncoso, IMSS, Ciudad de México, Mexico; ^4^Programa de Posgrado en Ciencias Biológicas, UNAM, Ciudad de México, Mexico; ^5^Unidad de Desarrollo e Investigación en Bioprocesos (UDIBI), Instituto Politécnico Nacional, Ciudad de México, Mexico; ^6^Departamento de Inmunología, Escuela Nacional de Ciencias Biológicas-Instituto Politécnico Nacional (ENCB-IPN), Ciudad de México, Mexico; ^7^Departamento de Farmacología, Centro de Investigación y de Estudios Avanzados del IPN (Cinvestav-IPN), Ciudad de México, Mexico

## Abstract

The development of cervical cancer (CeCa) is associated with high-risk human papilloma virus (HR-HPV) infections, mainly HPV-16, which is present in more than 50% of cases. The presence of immunosuppressive factors in the early stages of the disease is also strongly linked to CeCa progression. In this context, it is unknown whether ectonucleotidases CD39 and CD73, which are involved in the production of adenosine (Ado) that suppresses the specific antitumor immune response, are present in precursor lesions of CeCa. In this pilot study, we analyzed the presence of CD39 and CD73 and their capacity to generate Ado in 25 cervical samples from patients with grade 1 cervical intraepithelial neoplasms (CIN-1) and 25 samples from normal donors (NDs) free of HPV infection. Cells obtained from cervical samples of CIN-1 patients positive for HPV-16 showed higher CD39 and CD73 contents compared to samples obtained from CIN-1 patients negative for HPV-16 and NDs. Interestingly, solubilized cervical mucus from these patients also showed higher contents of soluble CD39 and CD73, which were associated with a greater capacity to produce Ado from the hydrolysis of adenosine triphosphate (ATP) and adenosine monophosphate (AMP). In addition, serum samples of these patients showed higher levels of TGF-*β* than those of CIN-1 patients negative for HPV-16 and ND. These results suggest that persistent infection with HR-HPV, mostly HPV-16, in CIN-1 patients may promote the expression of CD39 and CD73 through the production of TGF-*β* in precursor lesions to generate an immunosuppressive microenvironment and allow its progression to CeCa.

## 1. Introduction

Cervical cancer (CeCa) is the fourth cause of cancer death in women worldwide, accounting for more than 300,000 deaths per year, of which more than 80% occur in developing countries [1, 2]. Persistent infection due to human papilloma virus (HPV) is the main factor for developing CeCa. To date, more than 200 HPV genotypes have been identified, of which HPV-16, HPV-18, HPV-31, HPV-33, HPV-35, HPV-39, HPV-45, HPV-51, HPV-52, HPV-56, and HPV-58, which are considered high-risk HPV (HR-HPV), are associated with anogenital cancer [3–5].

Most HPV infections are eliminated by the immune system in approximately 1 or 2 years after exposure [6]. However, in rare cases, HPV can generate dysplastic changes in the cervix, known as cervical intraepithelial neoplasia (CIN), classified as CIN-1, CIN-2, or CIN-3 [7]. CIN lesions are clinically heterogeneous and may regress spontaneously or persist and progress to invasive cancer. The regression rates vary according to the degree, occurring in approximately 50-90% of women with CIN-1, 40% with CIN-2, and 30% with CIN-3 [8]. In this context, the immune system plays a significant role by eradicating HPV infection, even with established CIN; however, it has been proposed that the progression to CeCa is inevitably linked to an immunosuppressive microenvironment during carcinogenesis in the cervix [9]. Therefore, it is important to determine the molecular mechanisms involved in immunosuppression in the initial stages of CeCa.

Recent reports have shown that the adenosinergic pathway plays an important role in the pathogenesis of gynecological cancer [10]. In this pathway, Ado generated through the phosphohydrolysis of adenine nucleotides by the activity of CD39 ectoenzymes (ENTPD1, ectonucleoside triphosphate diphosphohydrolase-1, EC 3.6.1.5) and CD73 (5′-ectonucleotidase, EC 3.1.3.5) induces extracellular signaling through four Ado receptors (ARs: A1R, A2AR, A2BR, and A3R) coupled to G proteins and arranged in the membranes of target cells [11]. In the hypoxic tumor microenvironment, Ado exerts an immunosuppressive effect on the effector cells of the immune system by interacting with A2AR [12] in addition to participating in the promotion of tumor growth inducing proliferation, invasion, and metastasis of tumor cells [13, 14]. Our working group was the first to report that CeCa tumor cells positive for HPV-16 and HPV-18 produce higher levels of CD73 than those negative for HPV and strongly suppress the effector functions of cytotoxic T lymphocytes through the production of Ado. Furthermore, by silencing the E6 and E7 oncogenes in these tumor cells, the expression level of CD73 and its ability to produce Ado were strongly reduced, suggesting that HPV infection may favor the constitutive expression of CD73 in cervical neoplasms to contribute to the suppression of the immune response through the production of Ado [15]. To determine whether the presence of CD39 and CD73 is related to HPV infection in the early stages of CeCa, we analyzed the contents and hydrolytic activity of these ectonucleotidases in cervical samples from patients with CIN-1. For comparison purposes, cervical samples from NDs negative for HPV were also analyzed.

## 2. Methods

This was a cross-sectional, descriptive, and observational pilot study.

### 2.1. Biological Material

The study population was selected among patients who visited the Department of Gynecology of HGZ No. 2-A Troncoso, Mexican Social Security Institute (IMSS), Mexico City, Mexico. Cervical cytology samples were obtained between February 2016 and February 2018 after obtaining informed consent endorsed by the local bioethics committee. All women underwent cytological and histopathological analyses by means of a directed colposcopy. Two experienced cytotechnologists independently examined all Papanicolaou tests. Samples with an inconsistent diagnosis were excluded from the study. The cytology diagnoses were classified according to the Bethesda system, while a CIN-1 histopathological diagnosis was classified according to Richardt [16]. Women who were consistently negative to clinical and molecular tests were considered ND. Only CIN-1 patients who tested positive for HPV through polymerase chain reaction (PCR) were included in the study. Samples from patients with CIN-2 or CIN-3 were excluded from the study. Cervical cells were carefully collected by cleaning the ectocervix with a cytobrush (Cytobrush®, STERYLMEDICAL Co., Yangon, Myanmar). Samples were placed in a tube containing 2 mL of sterile phosphate-buffered saline (PBS) (Sigma-Aldrich, St. Louis, MO, USA) free of contamination to solubilize the mucus. Once the samples were centrifuged at 2000 rpm, the cell pellet was placed in a tube containing ThinPrep® PreservCyt® (Hologic Inc., Marlborough, MA, USA), while the supernatants were fractionated into tubes and stored at -20°C for the detection and determination of the hydrolytic activity of soluble CD39 and CD73. A portion of cells was used for immunocytochemical staining and another for further processing in TRIzol® (Thermo Fisher Scientific, Waltham, MA, USA). DNA was extracted from TRIzol® by the traditional phenol-chloroform technique. All samples were subjected to PCR molecular analysis, using the LINEAR ARRAY® HPV kit (Roche Diagnostics, CA, USA), for genotyping between 37 main types of HPV that infect the anogenital region (HPV-6, HPV-11, HPV-16, HPV-18, HPV-26, HPV-31, HPV-33, HPV-35, HPV-39, HPV-40, HPV-42, HPV-45, HPV-51, HPV-52, HPV-53, HPV-54, HPV-55, HPV-56, HPV-58, HPV-59, HPV-61, HPV-62, HPV-64, HPV-66, HPV-67, HPV-68, HPV-69, HPV-70, HPV-71, HPV-72, HPV-73, HPV-81, HPV-82, HPV-83, and HPV-84), following the supplier's instructions.

### 2.2. Detection of CD39 and CD73 in Cervical Cytologies

The expression of ectonucleotidases CD39 and CD73 in cervical cells was determined by immunocytochemical staining with a mouse anti-CD39 monoclonal antibody (clone eBioA1 (A1), 14-0399-82, eBioscience) (Thermo Fisher Scientific, Waltham, MA USA) and an anti-CD73 rabbit-derived polyclonal antibody (Cat. NBP1-85740) (Novus Biologicals, Cambridge, UK) according to protocols previously described [17, 18]. Briefly, 5 × 10^3^ cervical cells fixed in ThinPrep PreservCyt® solution were deposited onto positively charged glass slides (Kling-On HIER Slides, Biocare Medical). Subsequently, the cells were permeated with 0.01% Triton X-100 in PBS and incubated for 2 h with 2% (*w*/*v*) bovine serum albumin (BSA) in PBS (Sigma-Aldrich, St. Louis, MO, USA). Next, the cells were incubated for 1 h with the antibodies. After two washes with PBS, the slides were incubated for 1 h using goat anti-mouse or anti-rabbit secondary antibodies conjugated to horseradish peroxidase (Dako, Carpinteria, CA, USA). The development was carried out with a substrate-chromogen solution, 3,3'-diaminobenzidine dihydrochloride (DAB) (Sigma-Aldrich, St. Louis, MO, USA), for 3-5 min. Cells incubated with the correspondent secondary antibody were included as a control. Nuclei were stained with Mayer's hematoxylin (Sigma-Aldrich, St. Louis, MO, USA). The slides were scanned through an Aperio CS digital pathology device (San Diego, CA, USA) to obtain electronic files. The presence of proteins CD73 and CD39 in the cervical cells was determined by densitometric analysis considering the total expression density (TED) from 100 cells per sample using Image-Pro Plus version 6.0.

### 2.3. Detection of Soluble CD39 and CD73

The content of ectonucleotidases CD39 and CD73 solubilized in supernatants of cervical samples was determined by an enzyme-linked immunosorbent assay (ELISA). Data were interpolated in type curves of recombinant enzymes CD39 and CD73 (R&D Systems, Minneapolis, MN, USA) using different concentrations (1-100 ng/mL) diluted in PBS. To detect the content of soluble CD39 and CD73 in cervical samples, we previously determined the total protein concentration in each supernatant by using the Bradford reagent (Sigma-Aldrich, St. Louis, MO, USA). Afterwards, 2 *μ*g of total protein (in a final volume of 100 *μ*L) placed in triplicate in 96-well flat-bottomed plates for the ELISA/RIA (Corning Inc., USA) was incubated for 1 h at 37°C and then overnight at 4°C. Next, the plates were washed with wash solution (PBS-0.1% Tween-20) and then incubated with blocking solution (2% BSA *w*/*v* in PBS-0.1% Tween-20) for 2 h at 37°C. Once the plates were washed, anti-CD39 and anti-CD73 antibodies were added at a dilution of 1 : 500 in blocking solution and incubated for 2 h. The plates were washed six times and incubated with goat anti-mouse or anti-rabbit IgG antibodies bound to alkaline phosphatase (Thermo Fisher Scientific, Waltham, MA, USA) at a dilution of 1 : 5000. The plates were incubated at 37°C for an additional 2 h, and after eight washes, the alkaline phosphatase substrate (Sigma-Aldrich, St. Louis, MO, USA) was added to diethanolamine solution (Sigma-Aldrich, St. Louis, MO, USA) at 10% (pH 9.8). The reading was performed at a wavelength of 405 nm using an ELISA plate reader.

### 2.4. Hydrolytic Activity of Soluble CD39 and CD73

To determine the hydrolytic activity of ectonucleotidases CD39 and CD73 solubilized in cervical samples, 2 *μ*g of total protein from the supernatants of the samples collected was incubated with adenosine triphosphate (ATP) or adenosine monophosphate (AMP) at a final concentration of 5 mM. After incubation for 72 h, Ado production was evaluated. To inhibit the enzymatic activity of CD39 and CD73, the specific inhibitors sodium polyoxotungstate (POM-1) (Sigma-Aldrich, St. Louis, MO, USA) and adenosine 5′-(*α*,*β*-methylene)diphosphate (APCP) (Sigma-Aldrich, St. Louis, MO, USA), respectively, were added at a final concentration of 5 mM as previously described [19]. The total volume of each reaction was 50 *μ*L. The amount of Ado produced by each sample incubated with ATP or AMP was evaluated through high-performance liquid chromatography, applying 25 *μ*L of each reaction to a chromatograph (UPLC Acquity, Waters Corporation, Milford, MA, USA) using a mobile phase composed of 0.5% acetonitrile, 5% methanol, and 94.5% sodium acetate (0.25 M and pH 6.3). Prior to the reading, the samples were filtered in Amicon filters of 3000 Daltons (Millipore Corporation, USA). An Ado standard curve was prepared to evaluate the Ado content in the different samples using Empower 3 (Waters Corporation, Milford, MA, USA).

In some assays, different concentrations (5 mM, 0.5 mM, 0.05 mM, and 0.005 mM) of POM-I or APCP were used to inhibit the capacity of supernatants of cervical samples to hydrolyze ATP or AMP. The presence of the products (AMP and Ado) was detected by thin-layer chromatography (TLC) by placing 1 *μ*L of each supernatant on fluorescent gel-coated plates (Whatman, GE Healthcare, Freiburg, Germany). Samples were eluted for 1 h using a mobile phase composed of isobutanol : isoamyl alcohol : ethoxyethanol : ammonia : water (9 : 6 : 18 : 9 : 15) [20], and 5 mM AMP, Ado, and inosine (Ino) (Sigma-Aldrich) were used as standard controls. Compounds were visualized using an UV transilluminator.

### 2.5. Quantification of TGF-*β*1

To quantify TGF-*β*1 content in serum samples, the human TGF-*β*1 Quantikine ELISA Kit (R&D Systems) was used according to the manufacturer's protocol.

### 2.6. Statistical Analysis

All numerical data are presented as the mean value ± standard error of the mean (SEM) of three independent experiments. Comparisons and correlations were evaluated by multivariate statistical analysis using GraphPad Prism version 7 (La Jolla, CA, USA). Values < 0.05 were considered statistically significant.

## 3. Results

### 3.1. Participant Characteristics

The present study was performed using 50 cervical samples from women who visited the Department of Gynecology of HGZ Troncoso, IMSS, and according to the clinical assessment and histopathological study. Twenty-five samples corresponded to women whose colposcopy was positive for CIN-1 and 25 ND samples that were negative for HPV infection and whose colposcopy was negative for CIN-1. All samples and clinical data from the participants were taken after obtaining informed consent, in accordance with the ethics and confidentiality requirements related to working with human samples from the institutions involved. The mean age of the CIN-1 patients was 30.4 (range 19-43) years (Table 1), and that of the ND group was 29.9 (range 18-44) years (Table 2). The average number of sexual partners and pregnancies reported by CIN-1 patients was 2.4 (range 1-4) and 1.72 (range 1-4), respectively (Table 1), while that of the ND group was 1.08 (range 1-3) and 1.2 (range 1-3) (Table 2), respectively. All cervical samples from CIN-1 patients were positive for HPV infection. The most frequent genotypes were HPV-16 (11/25, 44%), HPV-58 and HPV-52 (7/25, 28%), HPV-53 and HPV-61 (4/25, 16%), and HPV-39 and HPV-54 (3/25, 12%) (Table 1). Fifteen samples presented infection with a single HPV genotype, of which 7 presented mono-HPV-16 infection (HPV-16), and 10 samples had coinfections (CI) by two or more different HPV genotypes, of which only 4 were coinfected with HPV-16 (HPV-16+CI). On the other hand, 14 of the 25 CIN-1 samples were negative for HPV-16 infection (HPV-16neg). It is important to mention that CIN-1 patients, positive for HPV-16, either by mono-HPV-16 or by coinfection with other HPV genotypes (HPV-16+CI), exhibited significantly a greater number of sexual partners (averages 3.4 and 2.5) and pregnancies (averages 2.4 and 2.5), respectively, than those of NDs, whose averages were 1 and 1.2, respectively (Table 3).

### 3.2. Cervical Cytologies of Patients with CIN-1 and HPV-16 Infection Showed High CD39 and CD73 Contents

**Figure 1:**
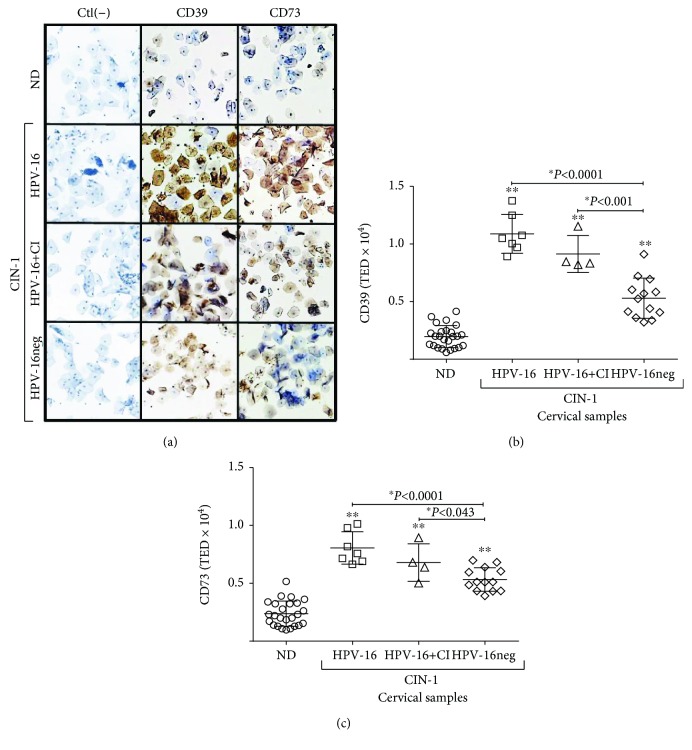
Expression of CD39 and CD73 in cervical cytologies of CIN-1 patients and normal donors (NDs). (a) Representative immunocytochemistry staining associated with the presence of ectonucleotidases CD39 and CD73 in cervical cytologies from NDs free of HPV infection and CIN-1 patients, positive either for HPV-16 (HPV-16) or for coinfection with HPV-16 and other HPV types (HPV-16+CI), or from HPV patients negative for HPV-16 (HPV-16neg) is shown in brown (10x magnification). Cells incubated only with the secondary antibody were included as a negative control (Ctl(-)). The presence of CD39 (b) and CD73 (c) proteins in cervical cytologies was analyzed through an Aperio AS device. The total expression density (TED) values are shown for each ND sample (circles), CIN-1 patients positive for HPV-16 (squares), patients with HPV-16+CI (triangles), and HPV-16neg patients (diamonds). ∗ indicates a statistically significant difference between groups. *P* values were calculated using the Wilcoxon signed-rank test and Student's *t*-test.

**Figure 2:**
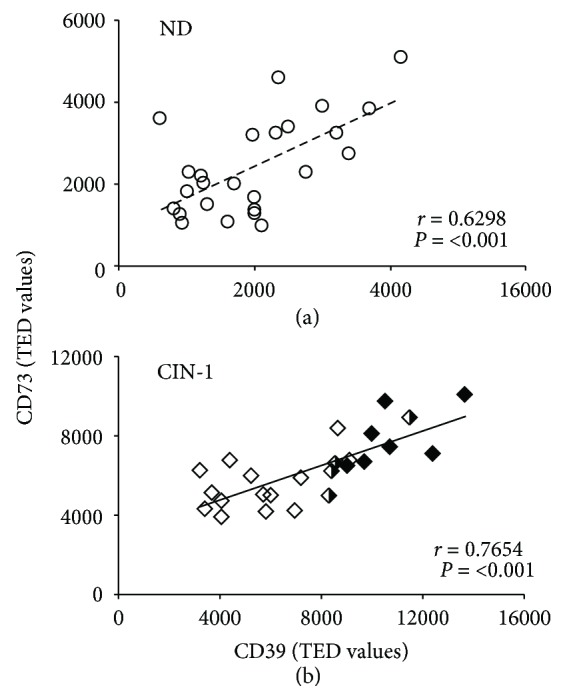
Correlation between the contents of CD39 and CD73 in the cytologies of CIN-1 patients and NDs. A positive (*r*) correlation between the TED values of CD39 and CD73 is shown for (a) NDs free of HPV infection (open circles) (*r* = 0.6298, *P* < 0.001) and (b) CIN-1 patients (*r* = 0.7654, *P* < 0.001). The contents of CD39 and CD73 are indicated in samples from CIN-1 patients positive for HPV-16 (HPV-16, black diamonds), patients with coinfection with HPV-16 and other HPV types (HPV-16+CI, black and white diamonds), and CIN-1 patients negative for HPV-16 (HPV-16neg, white diamonds). TED: total expression density.

The presence of CD39 and CD73 was visible mainly at the membrane and in the cytoplasm (Figure 1(a)). The TED for both ectonucleotidases was significantly higher in CIN-1 patients compared to NDs. The TED averages for CD39 and CD73 in CIN-1 patients were 7606 ± 597 and 6379 ± 343 pixels, respectively, while those in NDs were 1989 ± 194 and 2451 ± 234 pixels, respectively (Figures 1(b) and 1(c)). In fact, in the CIN-1 patients, those with HPV-16 and HPV-16+CI showed TED values of CD39 and CD73 that were higher than the values in HPV-16neg one. However, no differences of TED values of CD39 and CD73 were observed between HPV-16 and HPV-16+CI groups (Figure 1(b) and (c)). On the other hand, we observed a positive correlation between CD39 and CD73 contents in both the ND (0.6298, *P* < 0.001) (Figure 2(a)) and CIN-1 (0.7654, *P* < 0.001) groups (Figure 2(b)).

### 3.3. Presence of Soluble CD39 and CD73 in Cervical Samples from CIN-1 Patients and NDs

**Figure 3:**
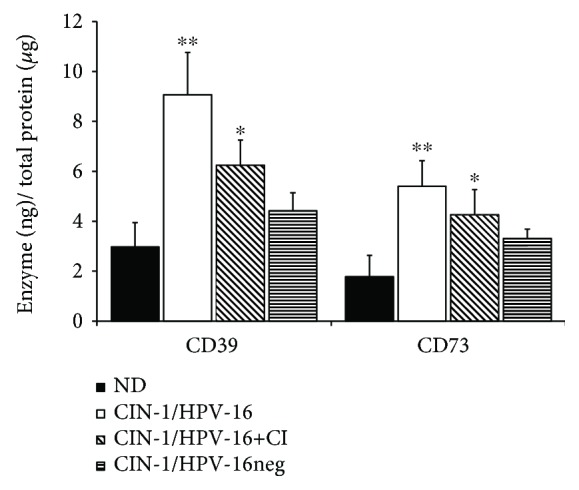
Contents of CD39 and CD73 in cervical samples from CIN-1 patients and NDs. The presence of CD39 and CD73 solubilized in cervical samples was determined by ELISA as described in Methods. The contents of CD39 and CD73 in cervical samples from NDs free of HPV infection (black bars) and from CIN-1 patients, positive either for HPV-16 (HPV-16, white bars) or for coinfection with HPV-16 and other HPV genotypes (HPV-16+CI, diagonal lines), or from HPV patients negative for HPV-16 (HPV-16neg, horizontal lines) are shown. Data represent three independent experiments, and the means ± SEM are shown. ∗ indicates a significant difference (*P* < 0.05) relative to the ND group. *P* values were calculated using the Wilcoxon signed-rank test and Student's *t*-test.

The expression of oncogenes E6 and E7 in cells positive for HR-HPV significantly increases protein secretion and extracellular microvesicles that may suppress the immune response [21–23]. Considering that cytological samples from CIN-1 patients had higher levels of CD39 and CD73 than ND samples, we detected the presence of these ectonucleotidases in the supernatants of the cervical samples using ELISA and type curves of recombinant CD39 and CD73. The supernatants of cervical samples from patients with CIN-1 positive for HPV-16, either by mono-HPV-16 or by coinfection with other HPV genotypes (HPV-16+CI), presented significantly higher contents (*P* < 0.05) of soluble CD39 and CD73 than those of ND samples. The contents of CD39 and CD73 in the supernatants of ND samples were 2.87 ± 0.98 and 1.71 ± 0.86 ng/*μ*g of total protein, respectively. Cervical samples from patients with CIN-1 positive for HPV-16, HPV-16+CI, and HPV-16neg had 8.84 ± 1.65 and 5.32 ± 0.99, 6.14 ± 0.92 and 4.2 ± 0.41, and 4.3 ± 0.76 and 3.24 ± 0.39 ng/*μ*g of total protein, respectively (Figure 3).

### 3.4. Cervical Samples from Patients with CIN-1 HPV-16+ Showed a High Capacity to Generate Adenosine by the Hydrolysis of ATP and AMP

**Figure 4:**
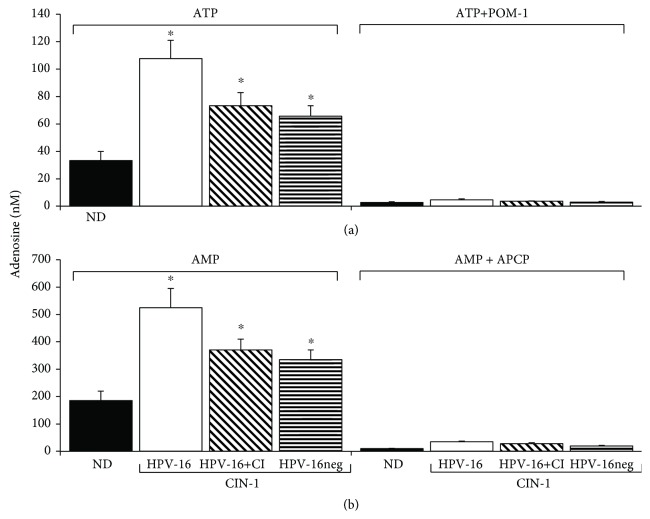
Hydrolytic activity of CD39 and CD73 solubilized in cervical samples from CIN-1 patients and NDs. Aliquots of 2 *μ*g of total protein obtained from the supernatants of cervical samples from NDs negative for HPV (ND, black bars) and CIN-1 patients, positive either for HPV-16 (HPV-16, white bars) or for coinfection with HPV-16 and other HPV types (HPV-16+CI, diagonal lines), or HPV patients negative for HPV-16 (HPV-16neg, horizontal lines) were incubated in the presence of 5 mM of ATP or AMP and in the presence or absence of POM-I or APCP, specific inhibitors of CD39 and CD73, respectively. After 72 h of incubation, the Ado product of ATP (a) or AMP (b) hydrolysis was evaluated by UPLC. ∗ indicates a significant difference (*P* < 0.01) of the Ado product of CIN-1 samples compared to NDs. Data represent three independent experiments.

**Figure 5:**
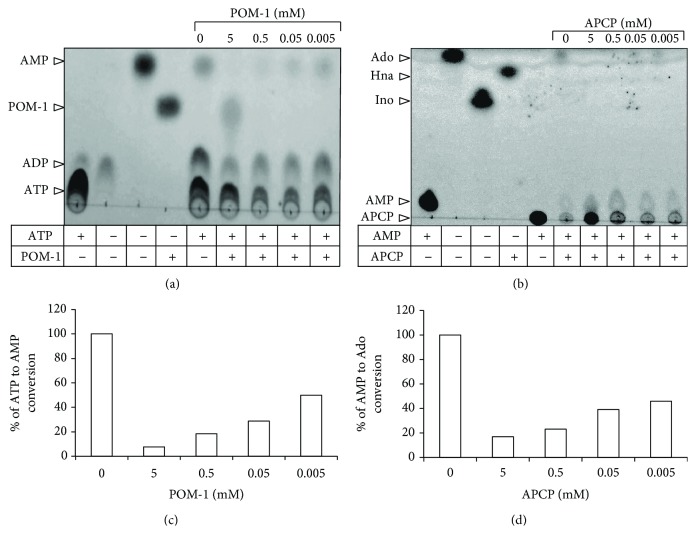
Effect of POM-1 and APCP to inhibit the capacity of supernatants of cervical samples from patients with CIN-1 HPV-16+ to hydrolyze ATP or AMP. Aliquots of 2 *μ*g of total protein obtained from the supernatants of cervical samples from patients with CIN-1 HPV-16+ were incubated in the presence of 5 mM of ATP or AMP and in the presence or absence of (5 mM, 0.5 mM, 0.05 mM, and 0.005 mM) of POM-I or APCP, specific inhibitors of CD39 and CD73, respectively. After 72 h of incubation, the ATP to AMP (a) and AMP to Ado (b) conversions were detected by TLC. The percentages of ATP to AMP (c) and AMP to Ado (d) conversions in the presence of several concentrations of POM-1 or APCP were determined by densitometric analysis in relation to the respective basal conversion (in the absence of the inhibitors), which was considered 100%. A representative assay from three independent experiments is shown.

To analyze the hydrolytic activity of ectonucleotidases CD39 and CD73 solubilized in the supernatants of cervical samples from patients with CIN-1 and NDs, we incubated 2 *μ*g of total protein of each supernatant in the presence of 5 mM ATP or AMP and in the presence or absence of POM-I or APCP, specific inhibitors of CD39 and CD73, respectively. Aliquots of the supernatants were taken at the beginning and after 72 h of incubation to evaluate Ado production by means of UPLC. Samples from CIN-1 patients exhibited a greater capacity to produce Ado than ND samples. Notably, samples from CIN-1 patients positive for HPV-16 had the highest capacity to hydrolyze ATP and AMP. The average Ado concentration produced by incubation of the supernatants from ND samples in the presence of ATP and AMP was 31.72 ± 7.8 and 181.16 ± 56.97 nM, respectively (Figures 4(a) and 4(b)). In samples from CIN-1 patients positive for HPV-16, HPV-16+CI, and HPV-16neg, the average Ado concentration was 106.14 ± 23.96, 71.5 ± 7.23, and 64.21 ± 13.41 and 515 ± 51.32, 357 ± 50.55, and 328.5 ± 63 nM, respectively (Figures 4(a) and 4(b)). On the other hand, the addition of 5 mM of POM-I or APCP, specific inhibitors of CD39 and CD73, respectively, decreased the capacity of the supernatants to hydrolyze ATP or AMP by more than 90% in all cases (Figure 4). Interestingly, the addition of doses lower than 5 mM (500 *μ*M, 50 *μ*M, and 5 *μ*M) of POM-1 or APCP in supernatants of patients positive for HPV-16, which showed the highest contents of CD39 and CD73, suppressed in a dose-dependent manner the conversion from ATP to AMP or AMP to Ado (Figures 5(c) and 5(d)). The inhibition obtained of more than 50% supports the specific effect of these inhibitors. These results suggest that HPV-16 infection in CIN-1 patients can lead to the generation of an immunosuppressive microenvironment due to its greater association with higher levels of ectonucleotidases CD39 and CD73 and, therefore, its greater capacity to produce Ado.

### 3.5. Serum Samples from Patients with CIN-1 Positive for HPV-16 Infection Showed Higher Concentration of TGF-*β*1 Compared with Those from CIN-1 Patients Negative for HPV-16 and NDs

**Figure 6:**
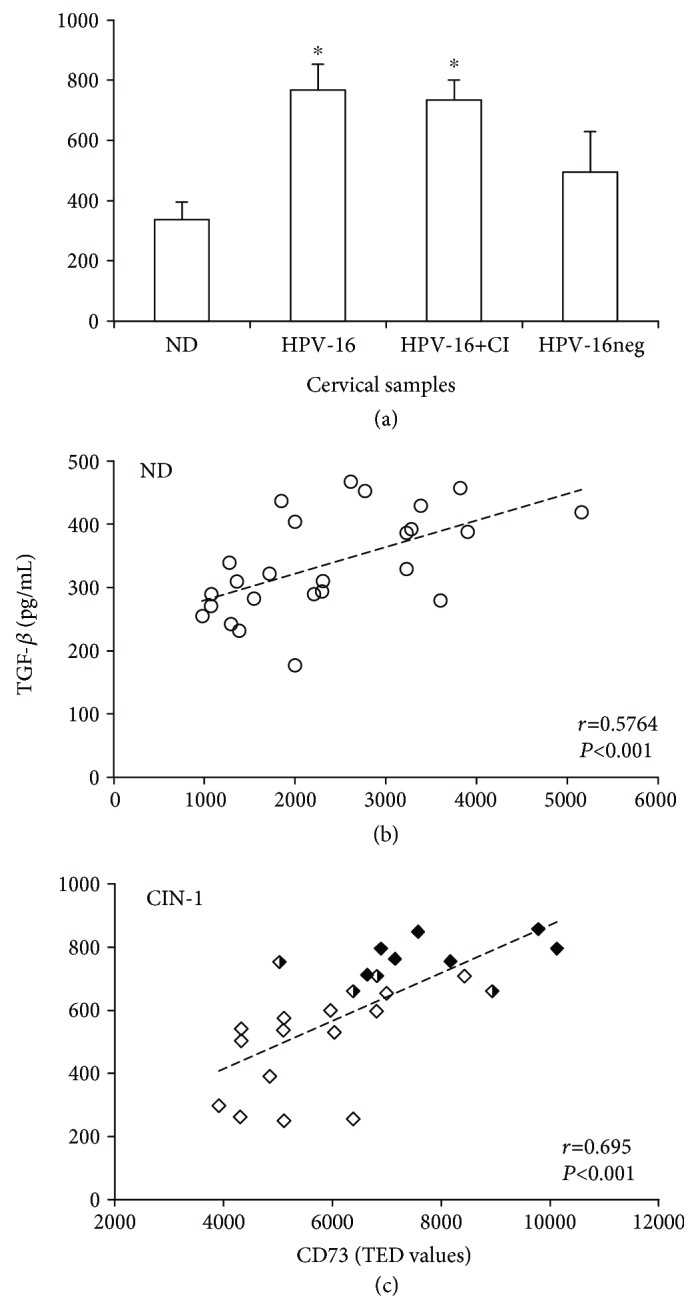
TGF-*β*1 content in serum samples from NDs and patients with CIN-1 and their correlation with the content of CD73 in their cervical samples. The content of TGF-*β*1 in serum samples derived from NDs and from CIN-1 patients positive for HPV-16, HPV-16+CI, and HPV-16neg is shown (a). Data are representative of three independent experiments, and the averages ± SEM are shown. ∗ indicates significant difference (*P* < 0.005) in the TGF-*β*1 concentration related to that of NDs. A positive (*r*) correlation between TGF-*β*1 and TED values of CD73 is shown for ND free of HPV infection (0.5764, *P* < 0.001) (b) and for CIN-1 patients (0.695, *P* < 0.001) (c). The contents of TGF-*β*1 and CD73 are indicated in samples from CIN-1 patients positive for HPV-16 (HPV-16, black diamonds), patients with coinfection with HPV-16 and other HPV types (HPV-16+CI, black and white diamonds), and CIN-1 patients negative for HPV-16 (HPV-16neg, white diamonds). TED: total expression density.

It has been suggested that TGF-*β*1 plays a key role in promoting human papillomavirus infection, as well as generating an immunosuppressive state in the local microenvironment of the cervix in HPV-infected women. Additionally, it has been reported that TGF-*β*1 levels increase in correlation with the severity of the lesions, and strong expression of this cytokine has been associated with poor survival in patients with CeCa [24–26]. Considering that we previously reported that TGF-*β*1 enhances CD73 expression in cervical cancer cells [27], we proceeded to analyze the levels of this cytokine in serum samples of the CIN-1 patients and NDs. Interestingly, patients positive for HPV-16, either by mono-HPV-16 or by coinfection with other HPV genotypes (HPV-16+CI), which showed the highest contents of CD73 in their cervical samples, also showed the highest levels of TGF-*β*1 (Figure 6). The average of TGF-*β*1 concentration contained in serum samples from NDs was 337 ± 59 pg/mL, while that from CIN-1 patients positive for HPV-16, HPV-16+CI, and HPV-16neg was 768 ± 85, 735 ± 65, and 494 ± 136 pg/mL, respectively (Figure 6(a)). In addition, we observed a positive correlation between CD73 and TGF-*β*1 contents in both the ND (0.5764, *P* < 0.001) (Figure 6(b)) and CIN-1 (0.695, *P* < 0.001) groups (Figure 6(c)).

## 4. Discussion

The development of CeCa is followed by several mechanisms of suppression and evasion of the immune response [28]. For example, during persistent infection with HR-HPV, the levels of viral proteins continue to be low, while capsid proteins are only expressed in the outer layers of the epithelium and, consequently, are out of reach of antigen-presenting cells [29]. Likewise, the pattern of Th1 cytokines, which are produced during the inflammatory state of infection and are important for the activation of CD4+ and CD8+ T lymphocytes and regression of the infection [30, 31], is inverted to a pattern of immunosuppressive cytokines, such as interleukin 10 (IL-10) and tumor growth factor beta (TGF-*β*), whose levels in tissues and plasma are directly correlated with the severity of the infection [32, 33], making it more likely to progress to CeCa [34, 35]. Additionally, it has been reported that proteins derived from high-risk HPVs, such as HPV-16, interfere with the immune response. For example, E6 and E7 proteins block IFN production by the infected cells [36] and reduce the expression of TLR9 [37] and cytokines, such as IL-8 [38] and IL-18 [39], which are proinflammatory molecules. Likewise, the proteins E5, E6, and E7 downregulate the expression of MHC class I molecules, reducing recognition of the HPV-infected cells by NK cells and by specific CTLs [40].

In turn, several studies have reported that the Ado produced by the functional activity of ectonucleotidases CD39 and CD73 participates in the suppression of the antitumor immune response and favors tumor progression in several types of cancer [14, 41–43]. We recently reported that HPV infection promotes the constitutive expression of CD73 in tumor cells of CeCa to contribute to the production of Ado and to inhibit the effector function of cytotoxic T lymphocytes [15]. However, it is unknown whether HPV infection is associated with the expression of CD39 and CD73 in precancerous lesions of the cervix. Therefore, in this study, we analyzed the hydrolytic activity of these ectonucleotidases in cervical samples from CIN-1 patients positive for HPV and compared them to cervical samples from NDs free of HPV infection. Interestingly, samples from CIN-1 patients, particularly those positive for HPV-16, as either mono- or coinfection with other HPV genotypes, showed higher levels of CD39 and CD73 than those from CIN-1 patients negative for HPV-16 and NDs. Likewise, we detected a greater amount of CD39 and CD73 solubilized in the supernatants of cervical samples from these patients, which is associated with a high capacity to produce Ado from ATP and AMP hydrolysis. TGF-*β* increases the levels of CD39 and CD73 in activated T cells and suppressive myeloid cells in both mice [44, 45] and humans [46, 47]. The expression of TGF-*β*1 in CeCa has been directly related to the degree of disease progression [24, 46] and to the expression of oncogenes E6 and E7 of HR-HPV [25]. In fact, oncogenes E6 and E7 of HPV-16 induce the activation of the human TGF-*β*1 promoter by recognizing the Sp1 sequence [48]. A recent study revealed that CeCa tumor cells infected with HR-HPV constitutively produce TGF-*β*, which is important for maintaining the expression of CD73 in tumor cells. We showed that the Ado product of the enzymatic activity of CD73 induced the production of TGF-*β* in tumor cells by interacting with A2AR and A2BR, suggesting an important connection between the adenosinergic pathway and TGF-*β* production in cells infected with HPV [25]. The transcriptional activation of CD39 and CD73 is also regulated by the transcription factor induced by hypoxia-inducible factor 1-alpha (HIF-1*α*) [49]. Therefore, TGF-*β*1 stabilizes HIF-1*α* [50]. Likewise, it has also been reported that oncogenes E6 and E7 of HPV-16 promote an increase in the expression of HIF-1*α* [51]. In the present study, high contents of ectonucleotidases CD39 and CD73 were found in the cervical samples from CIN-1 patients, positive for HPV-16, either by mono-HPV-16 or by coinfection with other HPV genotypes (HPV-16+CI). Interestingly, sera from these patients also showed the highest levels of TGF-*β*1; in consequence, it will be very interesting to know whether these contents of TGF-*β* are capable to inducing and maintaining CD39 and CD73 expression in the cervical microenvironment of CIN-1 patients HPV-16+. On the other hand, these patients exhibited significantly a greater number of sexual partners (averages 3.4 and 2.5) and pregnancies (averages 2.4 and 2.5), respectively, than NDs, whose averages were 1 and 1.2, respectively (Table 3). It is important to mention that high correlation between the number of sexual partners and CD39 and the number of pregnancies with the expression of CD39 and CD73 was observed in CIN-1 patients (Table 4). In fact, a large number of sexual partners and pregnancies have been reported as the main risk factors associated with persistent HPV infection and an increased risk of developing cervical dysplasia and cancer [52, 53]. Therefore, the present pilot study suggests that the production of TGF-*β*, associated with persistent infection with HPV-16, may induce and maintain the expression of the ectonucleotidases CD39 and CD73 and contribute to the generation of an immunosuppressive microenvironment in preneoplastic lesions of the uterine cervix and favor its progression. Due to the important role of the adenosinergic pathway in the suppression of the antitumor immune response [14, 41–43], its clinical relevance as a therapeutic target to several tumors, and the fact that CD73 expression in cervical cancer cells has been associated with increased metastatic potential [54], it would be interesting to repeat these experiments using a larger number of cervical samples from both patients with CIN-1 presenting infection with both low- and high-risk HPVs and including other lesional tissues, such as CIN-3 and cervical cancer. Consequently, it remains to be determined if CD39 and CD73 could be biomarkers in cervical cancer.

## 5. Conclusions

In this study, we provide evidence that cells obtained from cervical samples of CIN-1 patients positive for HPV-16 showed higher CD39 and CD73 contents compared to cells from samples of CIN-1 patients negative for HPV-16 and NDs. Interestingly, solubilized cervical mucus from these patients also showed higher contents of soluble CD39 and CD73, which were associated with a greater capacity to produce Ado from the hydrolysis of adenosine triphosphate (ATP) and adenosine monophosphate (AMP). In addition, serum samples of these patients showed higher levels of TGF-*β* than those of CIN-1 patients negative for HPV-16 and ND.

These results suggest that persistent infection with HR-HPV, mostly HPV-16, which is present in approximately 50% of CeCa cases [55, 56], may promote the expression of CD39 and CD73 through the production of TGF-*β* in precursor lesions to generate an immunosuppressive microenvironment and allow its progression to CeCa.

## Figures and Tables

**Table 1:** Clinical data of patients with CIN-1 and the HPV genotyping of their cervical samples.

Cervical sample number	HPV genotypes	Age (years)	Number of sexual partners	Number of pregnancies
1	16	36	4	3
2	16	29	2	2
3	16	43	3	2
4	16	32	3	2
5	16, 54, 56, 83	19	2	4
6	16	25	3	3
7	16	39	3	3
8	16	28	4	2
9	16, 33, 35, 52, 58, 61	27	3	2
10	16, 33, 35, 52, 58, 61	19	2	2
11	16,33, 35, 52, 58, 61	29	3	2
12	33, 35, 52, 54, 58, 62	31	1	2
13	2, 61	21	2	1
14	58	20	2	1
15	52	24	2	1
16	18	40	3	2
17	52	41	2	1
18	58	20	2	1
19	18, 54	37	2	0
20	39	42	2	1
21	53	33	2	1
22	39, 53, 70	26	1	1
23	33, 35, 52, 58, 59	34	3	1
24	53	27	2	1
25	39, 53, 70	39	2	2
Averages		30.4	2.4	1.72

**Table 2:** Clinical data of normal donors.

Cervical sample number	Age (years)	Number of sexual partners	Number of pregnancies
1	32	1	1
2	19	0	0
3	23	1	2
4	24	0	0
5	18	0	0
6	23	1	1
7	33	1	2
8	28	1	2
9	29	1	1
10	31	1	2
11	26	2	1
12	30	1	1
13	42	1	2
14	22	0	0
15	28	1	1
16	41	1	3
17	40	2	2
18	31	1	1
19	34	1	2
20	44	2	2
21	37	3	1
22	21	1	0
23	28	1	1
24	28	1	1
25	37	2	1
Averages	29.9	1.08	1.2

**Table 3:** Relation among clinical data of NDs and patients with CIN-1 with different HPV genotypes.

Clinical data	NDs	CIN-1 patients			
		HPV-16	HPV-16+CI	HPV-16neg	*P* values
Age (years)	29.96 ± 7.26	33.14 ± 6.46	23.5 ± 5.26	31.07 ± 8.07	—

Number of sexual partners	1.08 ± 0.7	3.4 ± 0.69^a^	2.5 ± 0.57^b^	2 ± 0.55^c^	<0.0001 vs. ND^a^ <0.0077 vs. ND^b^ <0.0002 vs. ND^c^

Number of pregnancies	1.2 ± 0.8	2.4 ± 0.53^d^	2.5 ± 1^e^	1.14 ± 0.53^f, g^	<0.0008 vs. ND^d^ <0.0077 vs. ND^e^ <0.0001 vs. HPV-16^f^ <0.002 vs. HPV-16+CI^g^

ND: normal donors; HPV-16: CIN-1 patients positive for HPV-16; HPV-16+CI: CIN-1 patients with coinfection with HPV-16 and other HPV types; HPV-16neg: CIN-1 patients negative for HPV-16. *P* values were calculated using the Wilcoxon signed-rank test and Student's *t*-test.

**Table 4:** Correlation analysis of the clinical data of normal donors and patients with CIN-1 and the expression of CD39 and CD73 in their cervical cytology.

Cell marker expression	Normal donors			CIN-1 patients			*P* values
	Age (years)	Number of sexual partners	Number of pregnancies	Age (years)	Number of sexual partners	Number of pregnancies	
CD39	0.052	0.258	0.177	0.156	0.435^a^	0.7^b^	<0.029^a^ <0.0001^b^ <0.027^c^ <0.0003^d^
CD73	0.26	0.439^c^	0.234	0.011	0.326	0.665^d^	

Values of Pearson's coefficient (*r*) are shown.

## Data Availability

The data used to support the findings of this study are available from the corresponding author upon request.

## Abbreviations

Ado: Adenosine
AMP: Adenosine monophosphate
APCP: 5′-(*α*,*β*-Methylene)diphosphate
ARs (A1R, A2AR, A2BR, and A3R): Adenosine receptors
ATP: Adenosine triphosphate
CeCa: Cervical cancer
CD39: Ectonucleoside triphosphate diphosphohydrolase-1
CD73: 5′-Nucleotidase
CI: Coinfections
CIN-1: Grade 1 cervical intraepithelial neoplasms
HR-HPV: High-risk human papillomavirus
NDs: Normal donors
PCR: Polymerase chain reaction
POM-1: Sodium polyoxotungstate
TED: Total expression density
TGF-*β*: Transforming growth factor-*β*
UPLC: Ultraperformance liquid chromatography.
